# Exosomal microRNA‐4661‐5p–based serum panel as a potential diagnostic biomarker for early‐stage hepatocellular carcinoma

**DOI:** 10.1002/cam4.3230

**Published:** 2020-06-14

**Authors:** Hyo Jung Cho, Geum Ok Baek, Chul Won Seo, Hye Ri Ahn, Suna Sung, Ju A Son, Soon Sun Kim, Sung Won Cho, Jeong Won Jang, Suk Woo Nam, Jae Youn Cheong, Jung Woo Eun

**Affiliations:** ^1^ Department of Gastroenterology Ajou University School of Medicine Suwon South Korea; ^2^ Department of Biomedical Sciences Ajou University Graduate School of Medicine Suwon South Korea; ^3^ Department of Internal Medicine College of Medicine The Catholic University of Korea Seoul Korea; ^4^ Liver Cirrhosis Clinical Research Center Seoul Korea; ^5^ Department of Pathology College of Medicine The Catholic University of Korea Seoul Republic of Korea; ^6^ Functional RNomics Research Center The Catholic University of Korea Seoul Republic of Korea; ^7^ Department of Biomedicine & Health Sciences Graduate School of Medicine The Catholic University of Korea Seoul Republic of Korea

**Keywords:** exosome, hepatocellular carcinoma, microRNA‐4661‐5p, sequencing, tumor marker

## Abstract

Currently, a reliable serum biomarker for hepatocellular carcinoma (HCC) has not been established, particularly for early‐stage HCC (single tumor < 2 cm). We aimed to investigate diagnostic serum exosomal microRNA (exo‐miR) panel for early‐stage HCC. Driver oncogenic miR (onco‐miR) candidates were selected by integrative analysis of miR expression profiles from three different RNA sequencing datasets of human HCC. Expressions of selected onco‐miRs in serum exosome were measured using quantitative real‐time PCR. Diagnostic performances of serum exo‐miRs for HCC were evaluated in the test cohort (N = 24) and validation cohort (N = 144). Serum exo‐miR panels were developed using a logistic regression model, and their diagnostic performance was evaluated. Six promising driver onco‐miRs, including miR‐25‐3p, miR‐140‐3p, miR‐423‐3p, miR‐1269a, miR‐4661‐5p, and miR‐4746‐5p, were identified by integrative analysis of three different RNA sequencing datasets. Among the six candidates, four serum exo‐miRs (miR‐25‐3p, miR‐1269a, miR‐4661‐5p, and miR‐4746‐5p) showed promising performance in the test cohort with area under the receiving operator curve (AUROC) >0.8. In our validation study, serum exo‐miR‐4661‐5p could diagnose HCC in all stages (AUROC = 0.917), even in early stage (AUROC = 0.923), with a greater accuracy than other candidate serum exo‐miRs and serum AFP. The panel composed of exo‐miR‐4661‐5p and exo‐miR‐4746‐5p was identified as the most accurate biomarker for early‐stage HCC (AUROC = 0.947, 95% confidence interval = 0.889‐0.980, sensitivity = 81.8%, and specificity = 91.7%). In conclusion, exo‐miR‐4661‐5p–based serum panel is a promising diagnostic marker for early‐stage HCC.

## Graphical abstract

The present study derived potential serum exosomal microRNA panels for hepatocellular carcinoma (HCC) using a systematic, genome‐wide biomarker discovery approach. Serum exosomal microRNA‐4661‐5p–based panel is a potent diagnostic biomarker for early stage HCC and could also be used as a prognostic indicator in patients with HCC.

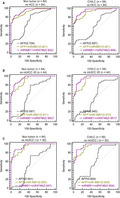

## 1 INTRODUCTION

Hepatocellular carcinoma (HCC) is the sixth most frequently diagnosed malignancy and third most common cause of cancer‐related mortality worldwide.^1^ Patients with early‐stage HCC (tumor size < 2 cm) could expect more than 70% of 5‐year survival rate after curative treatment.^2^ Conversely, patients with advanced‐stage HCC are eligible only for palliative treatments and show a poor prognosis with average survival of 1‐2 years.^3^ Therefore, detection of HCC at early stage is important to improve survival of patients. Liver professional societies, including the American Association for the Study of Liver Diseases (AASLD) and European Association for the Study of the Liver (EASL), recommend HCC surveillance every 6 months to detect HCC at early stages in patients with increased risk of developing HCC.^4^, ^5^, ^6^ Most of the practical guidelines recommend abdominal ultrasound (US) as a major HCC surveillance tool; however, the use of serum alpha‐fetoprotein (AFP) as an adjunct surveillance test remains controversial due to its low sensitivity and suboptimal cost‐effectiveness in detection of early‐stage HCC.^4^ Reliable serum biomarkers for early‐stage HCC are urgently required for improving the effectiveness of HCC surveillance program.

Recently, liquid biopsy has emerged as a highly promising technology to detect tumor‐derived circulating genetic molecules in blood.^7^, ^8^, ^9^ Exosomes are 30‐ to 100‐nm‐sized extracellular vesicles enclosing genetic materials of the parent (original) cell; they deliver the genetic material from the parent cell to the recipient cell.^10^, ^11^ Therefore, exosomes are considered a key player in intercellular communication,^12^ and exosomal contents are highlighted as major components of liquid biopsy analysis.^13^ Many studies have been performed to identify cancer cell‐specific exosomal contents to find novel efficient biomarkers and therapeutic targets. Among the exosomal contents, microRNAs (miRs) have attracted profound attention because increasing evidence indicates that the loading of specific miRs into exosome is an actively selected process governed by parent cell characteristics and not a random process.^14^, ^15^ Circulating miRs have been highlighted as potential biomarkers in HCC patients.^16^, ^17^, ^18^

In this study, we aimed to derive a reliable serum exosomal miRs (exo‐miRs) panel to diagnose HCC in early stages. To identify driver oncogenic microRNA (onco‐miR) candidates, systematic integrative analyses were performed using three different RNA sequencing datasets. Diagnostic effectiveness and clinical implications of the selected serum exo‐miR were evaluated in an independent validation cohort.

## 2 MATERIALS AND METHODS

### 2.1 Strategy to identify novel serum exo‐miRs for HCC

**Figure 1:**
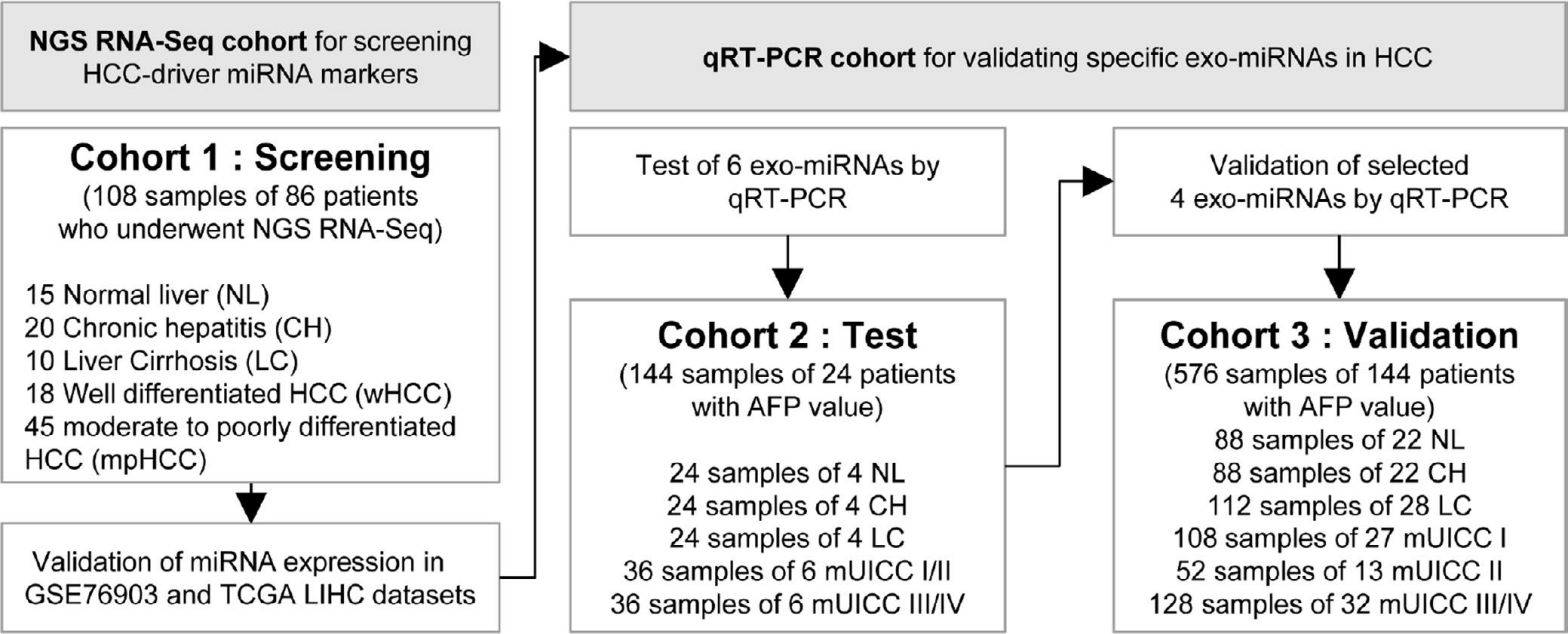
Strategy to identify novel serum exosomal microRNA for diagnosing HCC. AFP, alpha‐fetoprotein; CH, chronic hepatitis; HCC, hepatocellular carcinoma; LC, liver cirrhosis; miRNA, microRNA; mpHCC, moderate to poorly differentiated hepatocellular carcinoma; mUICC, modified Union for International Cancer Control; NGS, next‐generation sequencing; NL, normal; qRT‐PCR, quantitative real‐time polymerase chain reaction; RNA, ribonucleic acid; seq, sequencing; TCGA LIHC, The Cancer Genome Atlas Liver Hepatocellular Carcinoma; wHCC, well‐differentiated hepatocellular carcinoma

Figure 1 shows the strategy employed in the present study. Sequencing data from the Catholic University liver disease cohort were used to screen driver onco‐miR candidates (Cohort 1, screening cohort). Cohort 1 consisted of 108 snap‐frozen tissues from 86 subjects categorized based on their liver disease status as follows: 15 normal subjects, 20 with chronic hepatitis (CH), 10 with liver cirrhosis (LC), 18 with well‐differentiated HCC (Edmonson grade 1, wHCC), and 45 with moderate to poorly differentiated HCC (≥Edmonson grade 2, mpHCC). Expression of onco‐miRs selected in Cohort 1 was validated using two different public RNA sequencing (RNA‐seq) datasets from Tsinhua liver cancer hepatocellular carcinoma (LIHC) and the cancer genome atlas (TCGA) LIHC. The expressions of selected onco‐miRs in serum exosome were evaluated using qRT‐PCR in the Ajou university hospital liver disease cohort (720 serum samples from 168 subjects). The Ajou university hospital liver disease cohort was divided into two cohorts: Cohort 2 (test cohort) and Cohort 3 (validation cohort). Expression of onco‐miRs in serum exosome was tested in Cohort 2 (144 serum samples from 24 subjects). And, serum exo‐miRs with area under the receiving operator curve (AUROC) >0.8 in Cohort 2 were entered into the validation study using Cohort 3(576 serum samples from 144 subjects. AUROCs of the selected serum exo‐miRs were compared with those of serum AFP in the entire Ajou university hospital liver disease cohort. The serum panels using serum exo‐miRs and AFP were derived by logistic regression analysis, and the diagnostic performance of the selected serum panel was evaluated in Ajou university hospital liver disease cohort.

### 2.2 Clinical information of the cohorts and clinical term definitions

Detailed clinicopathological information of the Catholic university liver disease cohort is described in a previously published article.^19^ Baseline clinicopathological characteristics of cohorts 2 and 3 are detailed in Table 1. Serum samples and the corresponding clinical data, which were collected between January 2014 and December 2018, were obtained from the Biobank of Ajou university Hospital, a member of Korea Biobank Network. According to the definitions, the subjects were grouped into normal healthy individuals, patients with CH, patients with LC, and patients with HCC. Normal control was defined as 18‐ to 50‐year‐old subjects without any past medical history who visited the A Health Promotion Center for regular health check‐ups and showed completely normal health results. Patients with CH were defined as those with elevated aspartate aminotransferase (AST)/alanine aminotransferase (ALT) for more than six consecutive months. Patients with LC were diagnosed based on clinical findings, radiologic data, and/or histological findings.^20^ Diagnosis of HCC was determined according to the AASLD practice guidelines.^4^ Clinical data collected pertained to age, gender, etiology of liver disease, AST level, ALT level, platelet count, serum AFP level, serum albumin level, serum bilirubin level, and international normalized ratio (INR). Tumor size, tumor numbers, presence of vascular invasion, and tumor stage according to the modified Union for International Cancer Control (mUICC) staging system were investigated in HCC patients.^21^ Early‐stage HCC was defined as single tumor <2 cm in diameter, which was equivalent to mUICC stage I. High‐risk condition of developing HCC was defined as chronic hepatitis or LC. Disease‐free survival (DFS) was defined as the time from complete response after curative treatment to cancer recurrence. Overall survival (OS) was defined as the time from diagnosis of HCC to death from any cause.

**Table 1:** Baseline characteristics of the included patients

Variables	Cohort 2 (n = 24)			Cohort 3 (n = 144)			
	NL (n = 4)	CH (n = 4)	LC (n = 4)	HCC (n = 12)	NL (n = 22)	CH (n = 22)	LC (n = 28)	HCC (n = 72)
Age (y), mean ± SD	33.8 ± 4.1	44.8 ± 6.1	54.75 ± 13.40	53.4 ± 11.17	34.3 ± 7.7	45.0 ± 12.1	53.6 ± 10.4	55.1 ± 8.7
Male gender, n (%)	0 (0)	1 (25)	0 (0)	10 (83.3)	3 (13.6)	11 (50)	18 (64.3)	58 (80.6)
AST, IU/mL	18.00 ± 0.00	25.75 ± 10.34	65.75 ± 46.09	86.25 ± 139.29	16.36 ± 4.19	52.68 ± 55.25	87.11 ± 106.39	75.01 ± 95.24
ALT, IU/mL	11.33 ± 2.30	26.00 ± 14.02	52.75 ± 53.29	43.75 ± 34.07	14.09 ± 8.82	60.86 ± 70.27	80.82 ± 105.44	48.53 ± 61.82
Platelet, ×10^9^/L	335.00	180.33 ± 49.94	139.00 ± 120.95	189.42 ± 92.17	282.20 ± 34.65	192.95 ± 43.98	123.81 ± 72.12	157.79 ± 78.74
AFP (ng/mL), mean ± SD	1.80 ± 0.29	4.20 ± 1.59	14.7 ± 11.94	8705.94 ± 19 590.66	1.77 ± 0.79	13.90 ± 19.65	77.24 ± 142.09	4164.74 ± 14 646.97
Etiology, n (%)		4/0/0/0	4/0/0/0	12/0/0/0		21/1/0/0	21/3/2/0	65/3/3/1
HBV/HCV/alcohol/others								
Albumin (g/L), mean ± SD		4.73 ± 0.24	4.4 ± 0.54	4.06 ± 0.58		4.59 ± 0.38	3.93 ± 0.53	4.25 ± 0.54
Bilirubin (mg/dL), mean ± SD		0.55 ± 0.31	0.70 ± 0.29	0.71 ± 0.45		0.82 ± 0.31	1.16 ± 1.07	1.59 ± 4.25
INR, mean ± SD		1.09 ± 0.06	1.21 ± 0.02	1.09 ± 1.10		1.02 ± 0.05	1.24 ± 0.19	1.60 ± 2.13
Modified UICC stage, n (%)				3(25)/3(25)/2(16.7)/2(16.7)/2(16.7)				24(33.3)/14(19.4)/18(25.0)/10(13.9)/6(8.3)
I/II/III/IVa/Ivb								
Vascular invasion, n (%) Yes/No				5(41.7)/5(41.7)				24(33.3)/26(36.1)

Abbreviations: CH, chronic hepatitis; LC, liver cirrhosis; HCC, hepatocellular carcinoma; AST, aspartate transaminase; ALT, alanine transaminase; AFP, alpha‐fetoprotein; HBV, hepatitis B virus; HCV, hepatitis C virus; INR, international normalized ratio; UICC, Union for International Cancer Control.

This study was approved by the Institutional Review of Board (IRB) of Ajou university Hospital, Suwon, South Korea (AJRIB‐BMR‐KSP‐18‐397 and AJIRB‐BMR‐KSP‐18‐299). The need for informed consent was waived.

### 2.3 NGS RNA‐seq data analysis and publicly available genomic data analysis

For the large‐scale next‐generation sequencing RNA‐seq analysis, total RNA was extracted using TRIzol reagent (Invitrogen) from frozen liver tissues of Cohort 1 patients. RNA quality control was performed with the Agilent Bioanalyzer system (Agilent Technologies, Santa Clara, CA, USA). The library sequencing was followed by library quality check using the Agilent Bioanalyzer system. The sequencing was performed on Illumina HiSeq2000 machines (Illumina) using the standard Illumina protocol. All sequenced reads were checked for quality. To recapitulate the expression level of miRNA in HCC, genomic data were obtained from TCGA LIHC and the GEO database of the NCBI (Accession Numbers: GSE76903, Tsinghua LIHC). Level 3 miRNA expression data of TCGA LIHC miRNA‐seq V2 were log2 transformed [log2(TPM + 1)] and used to assess the gene expression levels.

### 2.4 Exosome isolation from the peripheral blood of patients

Serum was collected from the included subjects. It was aliquoted in 1 mL tubes and stored at −80°C for subsequent exosome isolation. Serum exosome was extracted using ExoQuick (System Biosciences). Exosomal RNA was isolated from the serum using SeraMir™ Exosome RNA Amplification Kit (System Biosciences).

### 2.5 Quantitative real‐time PCR (qRT‐PCR)

The expression of serum exo‐miRs was evaluated using qRT‐PCR. cDNA synthesis was performed using miScript RT II kit (QIAGEN). Furthermore, qRT‐PCR was performed using amfiSure qGreen Q‐PCR Master Mix and monitored in real time using CFX Connect Real‐Time PCR Detection System (Bio‐Rad Laboratories). miR‐1228‐3p was used as an internal control. The relative standard curve method (2^−ΔΔCT^) was used to determine the relative expression. The sequence of each miRNA was confirmed in miRBase database (http://www.mirbase.org; Table S1). All measurements were confirmed three times. Primer sequences used in the study are illustrated in Table S1.

### 2.6 Statistical analysis

All experiments were performed at least three times, and all samples were analyzed in triplicates. Data are presented as mean ± standard deviation (SD) or standard error of the mean (SEM). Statistical significance of the difference between experimental groups was assessed by paired or unpaired Welch's *t* test. IBM SPSS software version 22.0 (SPSS Inc) and GraphPad Prism version 7.01 were used for statistical analysis. Statistical significance was established at *P* < .05. Chi‐square test (two‐sided) was used to assess the association between categorical parameters. Survival curves were plotted using the Kaplan‐Meier method, and significant difference between the survival curves was determined using the Log‐rank test. Receiver operating characteristic (ROC) curves were analyzed to evaluate sensitivity, specificity, and respective AUROCs with 95% confidence interval (CI) for each candidate biomarker.

## 3 RESULTS

### 3.1 Identification of potential driver onco‐miRs by integrative analysis of sequencing data from three different RNA‐seq dataset

**Figure 2:**
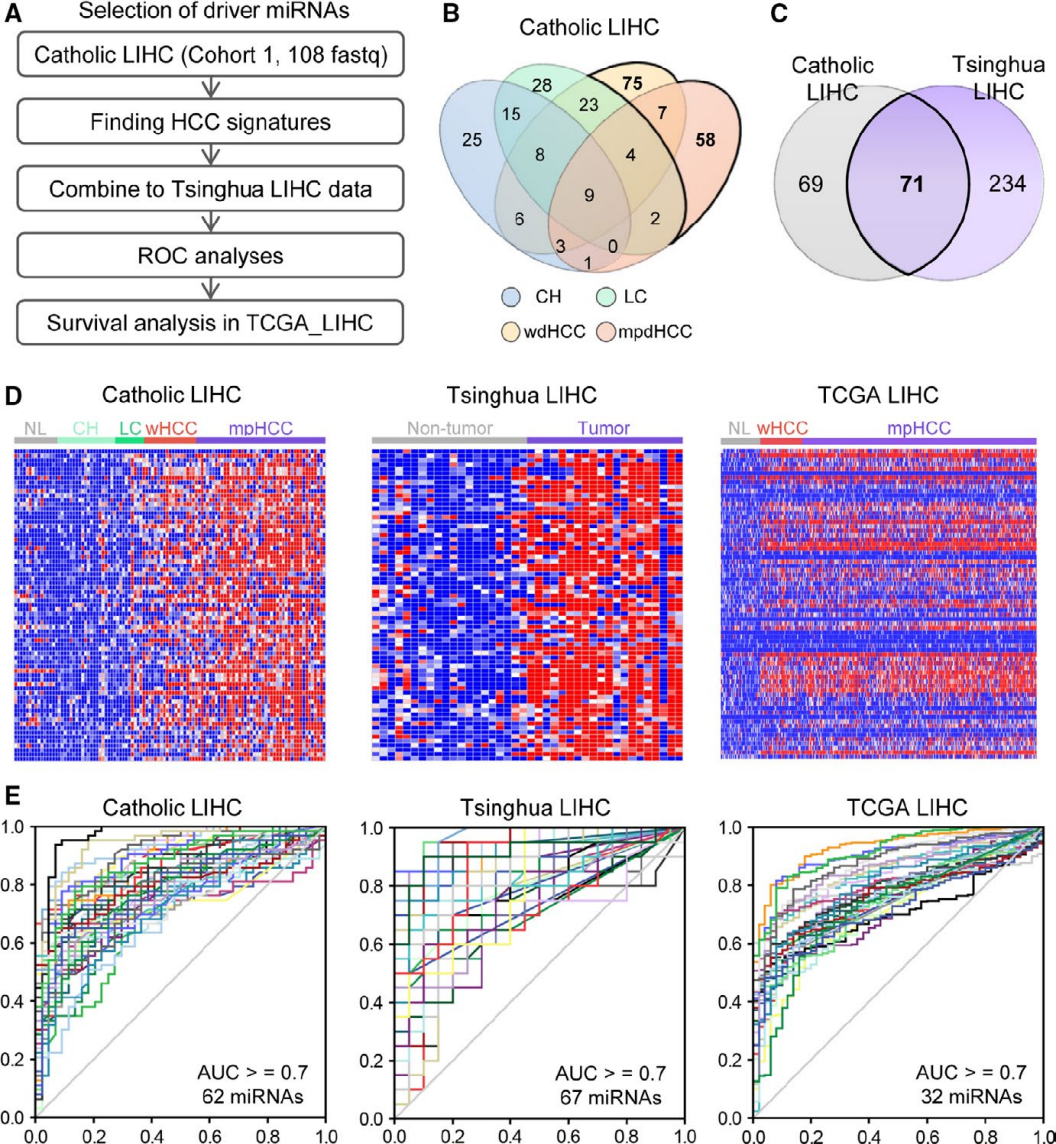
Integrative analysis of tissue sequencing data from three independent cohorts to identify onco‐miRs of HCC. A, Pipeline of gene expression analysis for identifying oncogenic miRs for HCC development. B, Venn diagram analysis of significantly overexpressed miRs in each disease status compared to normal subjects in Catholic LIHC cohort (Welch's t‐test, *P* < .05 and ≥1.3 fold). C, Venn diagram analysis to identify commonly overexpressed microRNAs in HCC tissues in Catholic LIHC and Tsinghua LIHC. D, Heatmaps of 71 microRNAs which were significantly overexpressed in HCC tissues in three different RNA sequencing datasets. E, Receiving operating curves of microRNAs with AUROC > 0.7 among 71 microRNAs in each HCC RNA sequencing datasets. CH, chronic hepatitis; HCC, hepatocellular carcinoma; LC, liver cirrhosis; LIHC, Liver Hepatocellular Carcinoma; miRNA, microRNA; mpHCC, moderate to poorly differentiated hepatocellular carcinoma; ROC, receiving operation curve; TCGA_LIHC, The Cancer Genome Atlas Liver Hepatocellular Carcinoma; wHCC, well‐differentiated hepatocellular carcinoma

To identify potential driver onco‐miRs, tissue‐small RNA sequencing data from Cohort 1 were analyzed, and HCC‐specific onco‐miR signatures were identified. To validate expression of selected miRs in Cohort 1, integrative analyses with two other different public RNA‐seq datasets, from Tsinghua LIHC and TCGA LIHC, were performed. Tsinghua LIHC data were used to verify expression of selected miRs, and TCGA LIHC data were used to select miRs associated with prognosis by performing survival analysis (Figure 2A).

**Figure 3:**
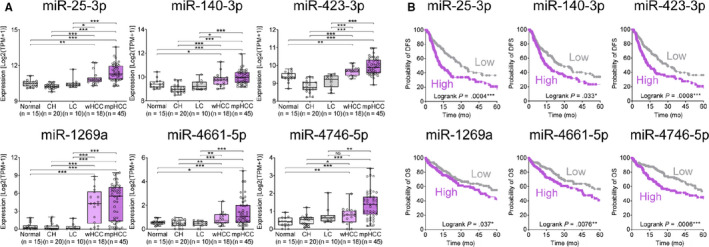
Comparison of miRs expression according to liver disease status and impact on survival. A, Expression of the selected six miRs according to liver disease status in Catholic LIHC cohort. B, Kaplan‐Meier survival analysis of HCC patients to compare overall survival and disease‐free survival according to the tissue expression of six miRs in TCGA LIHC dataset. CH, chronic hepatitis; HCC, hepatocellular carcinoma; LC, liver cirrhosis; LIHC, Liver Hepatocellular Carcinoma; miR, microRNA; mpHCC, moderate to poorly differentiated hepatocellular carcinoma; TCGA LIHC, The Cancer Genome Atlas Liver Hepatocellular Carcinoma; wHCC, well‐differentiated hepatocellular carcinoma

First, gene expression profiling of Cohort 1 sequencing data was performed. Significantly upregulated miRs compared to normal group (Welch's *t* test, *P* < .05 and ≥1.3 fold) was identified in each disease status. Figure 2B shows the distribution of upregulated miRs in a Venn diagram according to liver disease status compared to normal group. A total of 140 miRs were overexpressed at HCC status, but not overexpressed at nontumor status in Cohort 1. Tsinghua LIHC data were analyzed, and a total of 305 miRs (Welch's *t* test, *P* < .05 and ≥1.5 fold) overexpressed in HCC tissues when compared with nontumor tissues were identified. Integrative analysis of Cohort 1 and Tsinghua LIHC data revealed 71 commonly overexpressed miRs in HCC (Figure 2C). Figure 2D shows the heatmaps of the expressions of the 71 miRs in Cohort 1, Tsinghua LIHC, and TCGA LIHC. Figure 2E shows the receiving operator curves (ROCs) of the 71 miRs for diagnosing HCC in each cohort. AUROCs of 71 miRs were calculated, and the miRs with AUROC ≥ 0.7 in each cohort were selected for the next step (Figure 2E). Twenty‐six miRs, listed in Table S2, were identified as commonly overexpressed onco‐miRs in HCC tissues with AUROC > 0.7 in Cohort 1, Tsinghua LIHC, and TCGA LIHC (Figure S1). The 26 miRs were scored to evaluate potential as the driver onco‐miR. Table S2 enlists 26 miRs and their clinicopathological scores. The miRs associated with OS or DFS were assigned 1 point, and another 1 point was assigned for the overexpressed miRs in well‐differentiated HCC (Welch's *t* test, *P* < .05 and ≥1.5 fold), assuming that the driver onco‐miR is expressed early in differentiation phase. As a result, 6 of the 26 miRs, including miR‐25‐3p, miR‐140‐3p, miR‐422‐3p, miR‐1269a, miR‐4661‐5p, and miR‐4746‐5p, gained two points and were selected as potential driver onco‐miRs for next step. Figure 3A shows the expression of the six selected miRs according to liver disease status in the Cohort 1. The six selected miRs were significantly upregulated in HCC tissues compared to nontumor tissues (Welch's *t* test, *P < .05, **P < .01, ***P < .001). Figure 3B shows Kaplan‐Meier survival plot of HCC patients from TCGA LIHC dataset according to expression of six miRs. Patients with overexpressed miR‐25‐3p, miR‐140‐3p, and miR‐423‐3p showed significantly poor OS, whereas patients with overexpressed miR‐1269a, miR‐4661‐5p, and miR‐4746‐5p showed significantly shorter DFS (Log‐rank test, *P < .05, **P < .01, ***P < .001).

### 3.2 Expression of the selected six miRs in serum exosome: Test cohort

**Figure 4:**
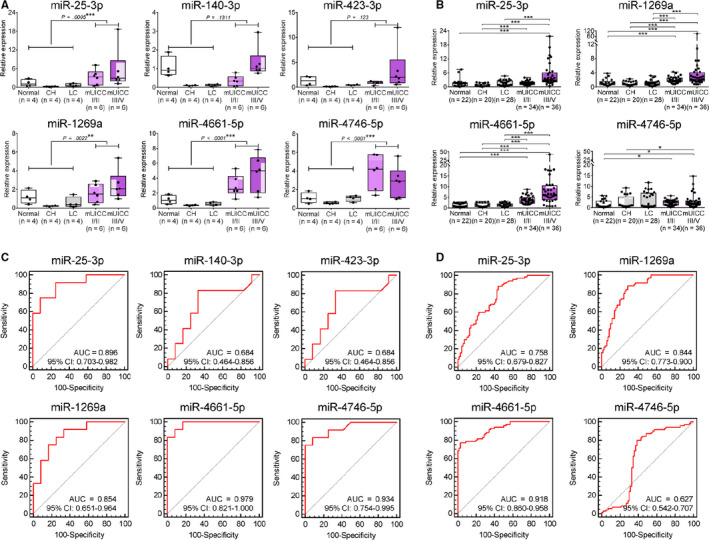
Expression according to liver disease status of the selected serum exo‐miRs and their diagnostic performance for diagnosing HCC. A, Expression of six serum exo‐miRs according to liver disease status in test cohort (Cohort 2). B, AUROC of six exo‐miRs for diagnosing HCC in test cohort (Cohort 2). C, Expression of four serum exo‐miRs according to liver disease status in validation cohort (Cohort 3). D, AUROC of four exo‐miRs for diagnosing HCC in validation cohort (Cohort 3). 95% CI, 95% confidence interval; AUC, the area under a receiver operating characteristic; AUROC, the area under a receiver operating characteristic; CH, chronic hepatitis; Exo‐miRs, exosomal microRNA; HCC, hepatocellular carcinoma; LC, liver cirrhosis; mUICC, modified Union for International Cancer Control

The expression of the six miRs in serum exosomes was evaluated in Cohort 2 (test cohort) to determine whether the selected miRs could be used as a serum biomarker for HCC. Cohort 2 included 24 subjects, including 4 normal subjects (n = 4), 4 patients with CH, 4 patients with LC, 6 patients with mUICCI/II, and 6 patients with mUICC III/IV. Figure 4A shows the expression value of the selected six serum exo‐miRs in Cohort 2. The expression of serum exo‐miR‐25‐3p, exo‐miR‐1269a, exo‐miR‐4661‐5p, and exo‐miR‐4746‐5p was significantly upregulated in the HCC group compared to nontumor status. The AUROCs were calculated, and four of the six miRs, including miR‐25‐3p, miR‐1269a, miR‐4661‐5p, and miR‐4746‐5p, showed AUROC > 0.8 (Figure 4B). Consequently, the four miRs with AUROC > 0.8 were entered into the validation study using Cohort 3.

### 3.3 Validation of the four serum exo‐miRs in Cohort 3

Cohort 3 included 144 subjects, comprising 22 normal subjects, 20 patients with CH, 28 patients with LC, 34 patients with mUICC I/II, and 36 patients with mUICC III/IV. The expression value of the selected four serum exo‐miRs—miR‐25‐3p, miR‐1269a, miR‐4661‐5p, and miR‐4746‐5p—was evaluated by qRT‐PCR. Figure 4C shows the expression values of the four serum exo‐miRs according to the disease status in Cohort 3. All four miRs were significantly upregulated in HCC patients compared to individuals with no tumor (Welch's *t* test, *P < .0 5, **P < .01, ***P < .001). Figure 4D shows ROCs of the four serum exo‐miRs for diagnosing HCC. Serum exo‐miR‐4661‐5p, with an AUROC value of 0.918 (95% CI: 0.860‐0.958), was identified as the most accurate diagnostic marker for HCC. The AUROC values of the other serum exo‐miRs were as follows: 0.758 for miR‐25‐3p (95% CI: 0.679‐0.827), 0.844 for miR‐1269a (95% CI: 0.773‐0.900), and 0.687 for miR‐4746‐5p (95% CI: 0.542‐0.707).

### 3.4 Comparison of diagnostic power of the selected four serum exo‐miRs and serum AFP

**Figure 5:**
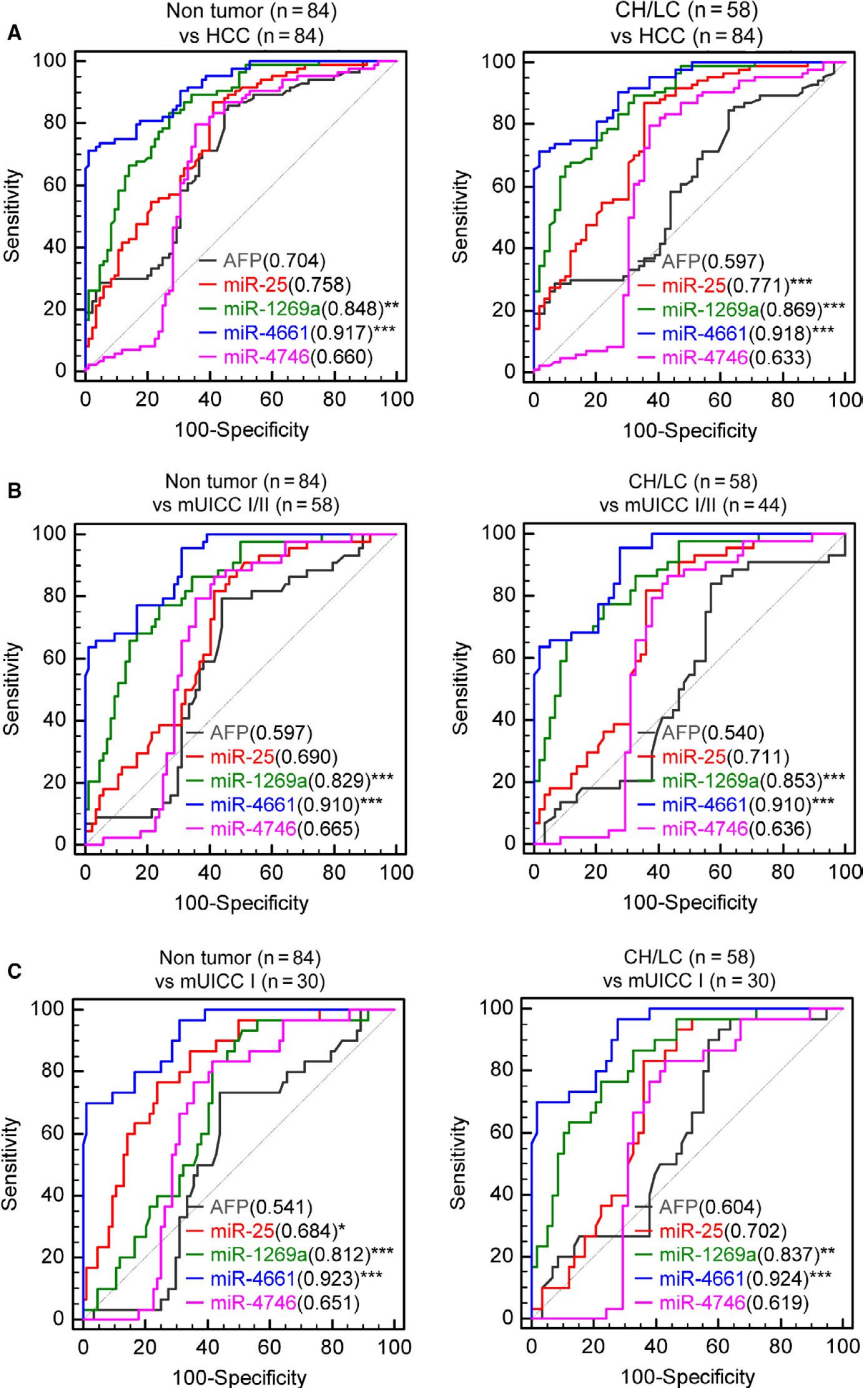
Diagnostic performance of serum exo‐miRs for diagnosing HCC in qRT‐PCR cohort. A, AUROCs for diagnosing HCC at all stages from nontumor subjects (normal, chronic hepatitis, and liver cirrhosis; left) and from patients at high risk of developing HCC (chronic hepatitis and liver cirrhosis; right). B, AUROCs for diagnosing HCC with mUICC stage I or II from nontumor subjects (normal, chronic hepatitis, and liver cirrhosis; left) and from patients at high risk of developing HCC (chronic hepatitis and liver cirrhosis; right). C, AUROCs for diagnosing HCC with mUICC stage I from nontumor subjects (normal, chronic hepatitis, and liver cirrhosis; left) and from patients at high risk of developing HCC (chronic hepatitis and liver cirrhosis; right). AFP, alpha‐fetoprotein; AUROC, the area under a receiver operating characteristic; CH, chronic hepatitis; Exo‐miRs, exosomal microRNA; HCC, hepatocellular carcinoma; LC, liver cirrhosis; miR, microRNA; mUICC, modified Union for International Cancer Control; qRT‐PCR, quantitative real‐time polymerase chain reaction

To compare the diagnostic power of serum AFP and the four selected exo‐miRs, AUROC of each serum marker was evaluated in the entire Ajou university hospital liver disease cohort (Figure 5). AUROC of AFP for HCC diagnosis was 0.704, whereas those of serum exo‐miR‐25‐3p, exo‐miR‐1269a, exo‐miR‐4661‐5p, and exo‐miR‐4746‐5p were 0.758, 0.848, 0.917, and 0.660, respectively. Serum exo‐miR‐1269a and miR‐4661‐5p showed significantly better performance than AFP (Figure 5A). In diagnosing HCC among the patients with CH and LC, serum exo‐miR‐25‐3p, miR‐1269a, and miR‐4661‐5p showed significantly greater AUROC values (0.690, 0.829, and 0.910, respectively) than serum AFP (0.597).

For distinguishing mUICC stage I/II HCC from nontumor status, serum exo‐miR‐1269a (AUROC = 0.853) and 4661‐5p (AUROC = 0.910) showed significantly greater AUROC values than serum AFP (0.597). For distinguishing mUICC stage I/II stage HCC from patients with CH or LC, serum exo‐miR‐1269a and 4661‐5p also showed greater AUROC value than serum AFP (AUROC; miR4661‐5p = 0.910, miR‐1269a 0.583, AFP = 0.540; Figure 5B).

Diagnostic accuracy of the serum exo‐miRs and AFP for diagnosing early‐stage HCC, which is equivalent to mUICC stage I, was also evaluated (Figure 5C). Serum exo‐miR‐25 (AUROC = 0.812), exo‐miR‐1269a (AUROC = 0.684), and exo‐miR‐4661‐5p (AUROC = 0.923) showed significantly better AUROC values than serum AFP (AUROC = 0.541). Subgroup analysis was performed to determine whether the candidate serum exo‐miRs could distinguish patients with early‐stage HCC from patients at high risk for developing HCC. Consequently, serum exo‐miR‐1269a (AUROC = 0.837) and exo‐miR‐4661 (AUROC = 0.924) showed significantly greater accuracy than serum AFP (AUROC = 0.604) in diagnosing early‐stage HCC among the patients with CH or LC.

### 3.5 Development of diagnostic panel for HCC

**Figure 6:**
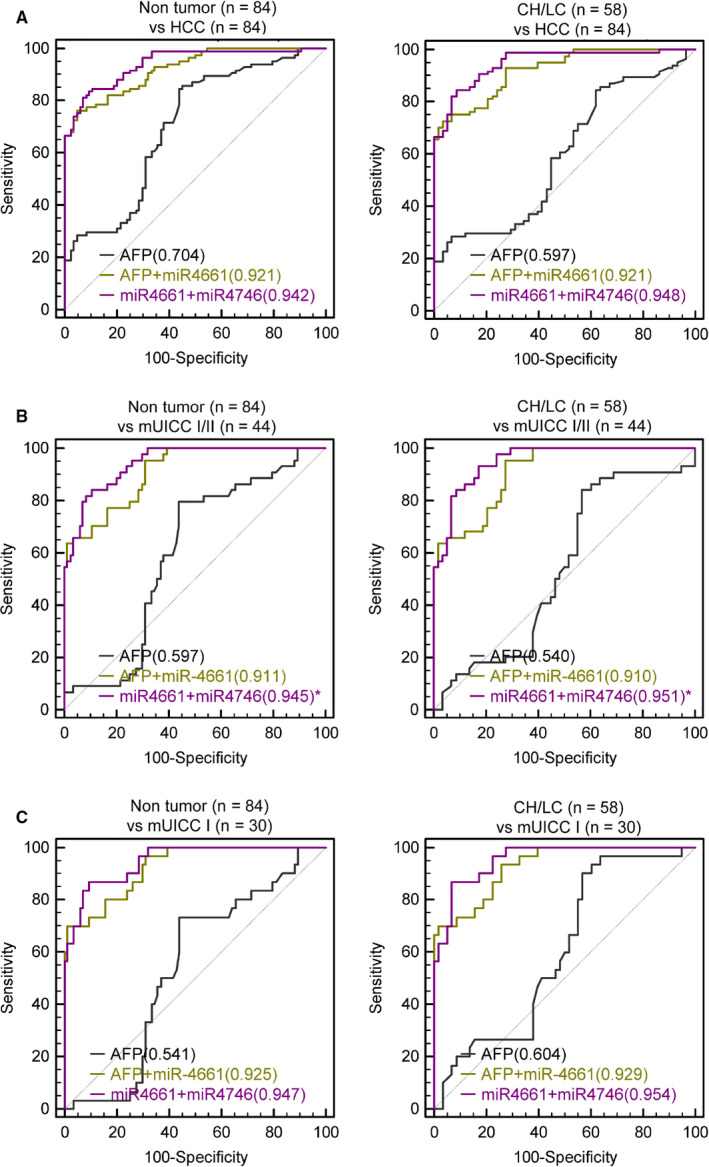
Diagnostic performance of the serum exo‐miR panels for HCC in qRT‐PCR cohort. A. AUROCs for diagnosing all stage HCC from nontumor subjects (normal, chronic hepatitis, and liver cirrhosis; left) and from patients at high risk of developing HCC (chronic hepatitis and liver cirrhosis; right). B, AUROCs for diagnosing HCC with mUICC stage I or II from nontumor subjects (normal, chronic hepatitis, and liver cirrhosis; left) and from patients at high risk of developing HCC (chronic hepatitis and liver cirrhosis; right). C, AUROCs for diagnosing HCC with mUICC stage I from nontumor subjects (normal, chronic hepatitis, and liver cirrhosis; left) and from patients at high risk of developing HCC (chronic hepatitis and liver cirrhosis; right). AFP, alpha‐fetoprotein; AUROC, the area under a receiver operating characteristic; CH, chronic hepatitis; Exo‐miRs, exosomal microRNA; HCC, hepatocellular carcinoma; LC, liver cirrhosis; miR, microRNA; mUICC, modified Union for International Cancer Control; qRT‐PCR, quantitative real‐time polymerase chain reaction

To derive most potent diagnostic panel, various combinations of the four serum exo‐miRs and serum AFP were attempted. Panels were made using linear logistic regression. The panel configuration was limited to two biomarkers considering their usability in clinical practice. The list of derived panels and their AUROCs is shown in Table S3. The cut‐off values of derived panels for measuring sensitivity and specificity are listed in Table S4. Cut‐off values for sensitivity and specificity were determined at the maximum Youden index. The combination of serum exo‐miR‐4661 and exo‐miR‐4746 showed most efficient diagnostic power for diagnosing HCC (AUROC = 0.942, 95% CI = 0.908‐0.975; Panel A). The second‐best combination was of serum AFP and serum exo‐miR‐4661 (AUROC = 0.921, 95% CI = 0.821‐0.927; Panel B). Table 2 demonstrates the comparison of the diagnostic performance of Panel A, Panel B, and serum AFP in various situations. Figure 6 shows the comparison of ROC curves between the derived serum panels and serum AFP (Figure 6A‐C). Panels A and B showed significantly superior performance to serum AFP in all situations (*P* < .0001). To diagnose early‐stage HCC, the AUROC of Panel A was measured as 0.947 (95% CI = 0.889‐0.980), with a sensitivity of 81.8% and specificity of 91.7% (Figure 6C, left). In addition, in patients at high risk for HCC, Panel A could distinguish early‐stage HCC with great accuracy (AUROC = 0.954, sensitivity = 86.7%, specificity = 93.1%; Figure 6C, right). Panel B also showed good diagnostic performance for early‐stage HCC in all included patients (AUROC = 0.925, 95% CI = 0.861‐0.966, sensitivity = 86.7%, specificity = 93.1%) for distinguishing early‐stage HCC patients from those with high risk for developing HCC including CH and LC (AUROC = 0.929, 95% CI = 0.854‐0.973, sensitivity = 70.0%, specificity = 98.3%).

**Table 2:** The area under the receiving operator curves, sensitivity, and specificity of the derived serum panels and serum AFP for diagnosing HCC

	*P* vs AFP	AUC	95% CI	Sensitivity (%)	Specificity (%)	PPV (%)	NPV (%)
HCC vs Nontumor							
AFP (20 ng/mL)	1	0.704	0.629‐0.772	38.095	71.429	57.143	53.571
AFP + miR‐4661‐5p	<.0001	0.921	0.870‐0.957	76.190	95.238	94.118	80.000
miR‐4661‐5p + miR‐4746‐5p	<.0001	0.942	0.895‐0.972	84.524	89.286	88.750	85.227
mUICC I and II vs Nontumor							
AFP (20 ng/mL)	1	0.597	0.507‐0.683	38.095	58.621	57.143	39.535
AFP + miR‐4661‐5p	<.0001	0.911	0.847‐0.954	72.619	96.552	96.825	70.886
miR‐4661‐5p + miR‐4746‐5p	<.0001	0.945	0.890‐0.977	84.524	91.379	93.421	80.303
mUICC I vs Nontumor							
AFP (20 ng/mL)	1	0.541	0.445‐0.634	15.909	71.429	22.581	61.856
AFP + miR‐4661‐5p	<.0001	0.925	0.861‐0.966	95.455	69.048	61.765	96.667
miR‐4661‐5p + miR‐4746‐5p	.95	0.947	0.889‐0.980	81.818	91.667	83.721	90.588
HCC vs CH and LC							
AFP (20 ng/mL)	1	0.597	0.511‐0.678	15.909	58.621	22.581	47.887
AFP + miR‐4661‐5p	<.0001	0.921	0.863‐0.959	95.455	72.414	72.414	95.455
miR‐4661‐5p + miR‐4746‐5p	<.0001	0.948	0.898‐0.979	93.182	82.759	80.392	94.118
mUICC I and II vs CH and LC							
AFP (20 ng/mL)	1	0.540	0.439‐0.639	10.000	71.429	11.111	68.966
AFP + miR‐4661‐5p	<.0001	0.910	0.837‐0.958	70.000	98.810	95.455	90.217
miR‐4661‐5p + miR‐4746‐5p	<.0001	0.951	0.889‐0.984	86.667	90.476	76.471	95.000
mUICC I vs CH and LC							
AFP (20 ng/mL)	1	0.604	0.494‐0.707	10.000	58.621	11.111	55.738
AFP + miR‐4661‐5p	<.0001	0.929	0.854‐0.973	70.000	98.276	95.455	86.364
miR‐4661‐5p + miR‐4746‐5p	<.0001	0.954	0.887‐0.987	86.667	93.103	86.667	93.103

Abbreviations: AFP, alpha‐fetoprotein; CH, chronic hepatitis; HCC, hepatocellular carcinoma; LC, liver cirrhosis; miR, microRNA; mUICC, modified Union for International Cancer Control.

### 3.6 Prognostic implication of the four serum exo‐miRs

**Figure 7:**
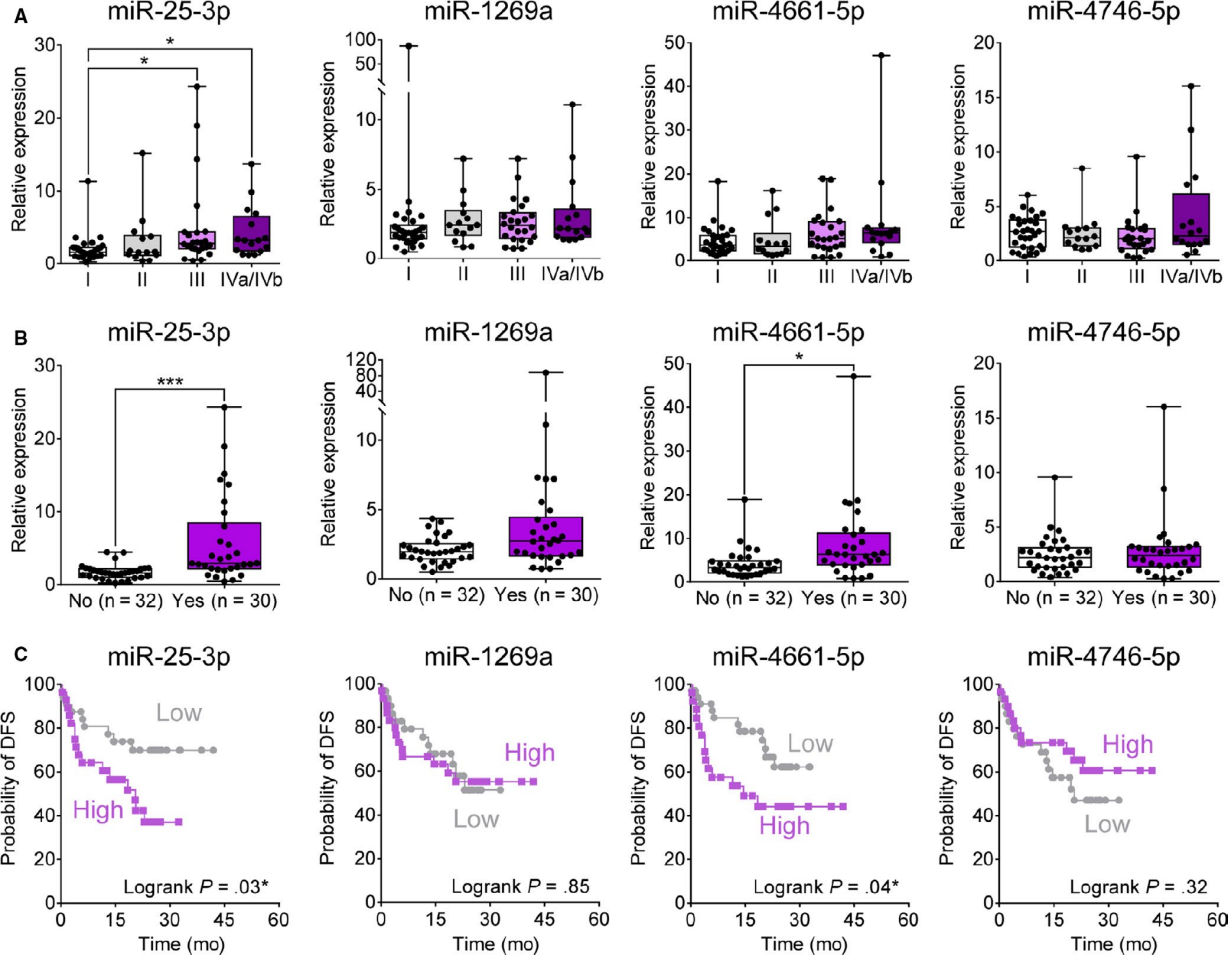
Prognostic significance of the 4 serum exo‐miRs in Ajou university hospital liver disease cohort. A, Expression of the serum exo‐miRs according to mUICC stage guidelines. B, Expression of the serum exo‐miRs according to vascular invasion. C, Disease‐free survival according to the expression of the four serum exo‐miRs in the qRT‐PCR cohort. Exo‐miR, exosomal microRNA; HCC, hepatocellular carcinoma; miR, microRNA; mUICC, modified Union for International Cancer Control; qRT‐PCR, quantitative real‐time polymerase chain reaction

Prognostic implications of the selected four serum exo‐miRs were evaluated. Serum exo‐miR‐25‐3p expression was found to be gradually increased with tumor stage advancement with statistical significance (Figure 7A). Upregulation of serum exo‐miR‐25‐3p and exo‐miR‐4661‐5p was significantly associated with vascular invasion (*P* = .05; Figure 7B). Kaplan‐Meier analyses were performed to identify prognostic implications of serum exo‐miRs. The patients were divided according to exo‐miR expression value: high expression group and low expression group. Cut‐off values were determined at median expression value of each exo‐miRs. The patients in the high expression group of serum exo‐miR‐25‐3p and serum exo‐miR‐4661‐5p showed significantly poor DFS compared to patients in the low expression group (Figure 7C).

## 4 DISCUSSION

This study derived novel and potent diagnostic serum exo‐miR panels for early‐stage HCC by integrative analysis of three different RNA‐seq datasets and serum exo‐miR expression data. The panels based on serum exo‐miR‐4661‐5p showed potential diagnostic effectiveness for HCC even in early stages with AUROC > 0.9. To our knowledge, this is the first study to identify serum exo‐miR‐4661‐5p–based panel as a potential diagnostic biomarker for early‐stage HCC.

In previous decades, several studies have showed that exo‐miRs from tumor cells are secreted into systemic circulation; therefore, circulating exo‐miRs have been investigated to develop ideal biomarkers and therapeutic targets in various intractable diseases.^22^, ^23^, ^24^ However, the results of previous studies regarding circulating exo‐miRs have not met expectations probably because they mainly focused on tissue expression data rather than serum data; however, tissue miR expression profile is somewhat different from circulating exo‐miRs expression profiles.^25^, ^26^ Therefore, a comprehensive and systematic genome‐wide biomarker discovery approach was planned in this study to find potential driver onco‐miRs which are commonly overexpressed in both HCC tissue and serum exosome. First, we performed integrative analysis of miR expression profile and clinicopathological data of human multistage HCC from three independent RNA‐seq datasets to identify potent driver onco‐miRs. Consequently, four miRs, including miR‐25‐3p, miR‐1269a, miR‐4661‐5p, and miR‐4746‐5p, were identified as commonly overexpressed miRs in both tissue and serum exosome in patients with HCC. We validated these findings and identified that serum exo‐miR‐4661‐5p–based panels were potent diagnostic serum biomarkers for early‐stage HCC. Systematic integrative analysis and validation strategies were the strength of the present study, which led to the successful development of a promising, novel serum exo‐miR panel for HCC.

Diagnosis of HCC at early stages is essential to improve the prognosis of patients with HCC.^27^, ^28^ Abdominal US with or without serum AFP is currently the main modality of HCC surveillance despite its sensitivity for early‐stage HCC being only 40%‐50%.^29^, ^30^ Recently, a meta‐analysis reported that concomitant use of serum AFP with US could improve the sensitivity for early‐stage HCC from 45% to 63%; however, the role of serum AFP for surveillance of early‐stage HCC remains controversial.^31^, ^32^ Therefore, many studies have been performed to find a reliable serum biomarker for early‐stage HCC; however, no biomarker better than serum AFP has been identified.^33^, ^34^ A reliable serum biomarker for early‐stage HCC is required to improve HCC surveillance. In this study, we tried to find a reliable biomarker of HCC, and serum exo‐miR‐4661‐5p was identified as an effective biomarker for HCC at all stages, including early stage, with a greater degree of accuracy than serum AFP (AUROC of serum exo‐miR‐4661‐5p = 0.923 vs AUROC of serum AFP = 0.541, *P* < .0001). In the present study, the sensitivity of serum AFP for early‐stage HCC was only about 10%–15%, whereas that of exo‐miR‐4661‐5p–based serum panel was about 80%‐95% (Table 2). Based on these findings, we can suggest exo‐miR‐4661‐5p–based serum panel as a more potent and reliable serum biomarker than serum AFP alone for diagnosing early‐stage HCC. Exo‐miR‐4661‐5p–based serum panel may improve the performance of HCC surveillance system in clinical practice. Further external validation in a larger cohort is required to incorporate it in real clinical practice.

To our knowledge, there has been no research regarding the clinical implication and mechanism of miR‐4661‐5p in patients with HCC. This study revealed the clinical implications of serum exo‐miR‐4661‐5p for the first time. It was revealed as a potent diagnostic serum marker of HCC and was also associated with the prognosis of patients with HCC; higher level of serum exo‐miR‐4661‐5p was correlated with poor prognosis of HCC patients. However, the action mechanism of miR‐4661‐5p was not evaluated in this study. Several previous studies have showed that miR‐4661 is a positive regulator of interleukin‐10 (IL‐10) expression in several inflammatory diseases.^35^, ^36^ IL‐10 is an anti‐inflammatory cytokine which is encoded by the *IL10* gene.^37^ Many studies have reported upregulated serum IL‐10 levels and higher expression of *IL10* gene in tissues of patients with HCC compared to non‐tumor status.^38^, ^39^ In addition, higher IL‐10 serum level is reportedly inversely correlated with the prognosis of patients with HCC as IL‐10 is a suppressor of antitumor immunity in HCC.^40^ Based on the results of previous studies and the present study, miR‐4661‐5p would have an oncogenic role in HCC, and it may suppress the antitumor immunity by upregulating the IL‐10 level. Further study is required to elucidate the action mechanism of serum exo‐miR‐4661‐5p in HCC and its correlation with IL‐10 expression levels.

In conclusion, the present study derived potential serum exo‐miR panels for HCC using a systematic, genome‐wide biomarker discovery approach. Serum exo‐miR‐4661‐5p–based panel is a potent diagnostic biomarker for early‐stage HCC and could also be used as a prognostic indicator in patients with HCC. Further mechanism study of exo‐miR‐4661‐5p in HCC and validation study in a large independent external cohort are required.

## CONFLICT OF INTEREST

The authors declare no conflicts of interest.

## AUTHOR CONTRIBUTION

Hyo Jung Cho: Drafting the manuscript and substantial contributions to the conception and design of the work; Geum Ok Baek, Chul Won Seo, Hye Ri Ahn: Substantial contributions to the acquisition of experimental data; Suna Sung and Ju A Son: acquisition of experimental data for revision; Jeong Won Jang: Substantial contributions to the acquisition of clinical data; Soon Sun Kim and Sung Won Cho: Study supervision; Jae Youn Cheong and Suk Woo Nam: Critical supervision; Jung Woo Eun: Study concept and substantial contributions to the analysis and interpretation of data for work and drafting the manuscript.

## Supporting information

Fig S1

Tabel S1

Table S2

Tabel S3

Table S4

## Data Availability

The datasets used and/or analyzed during the current study are available from the corresponding author on reasonable request.
