# Supplementary figures and images for: Exosomal microRNA‐4661‐5p–based serum panel as a potential diagnostic biomarker for early‐stage hepatocellular carcinoma
Source: Cancer Med. 2020 Jun 14;9(15):5459–72. doi: 10.1002/cam4.3230 (PMC7402848; doi:10.1002/cam4.3230)

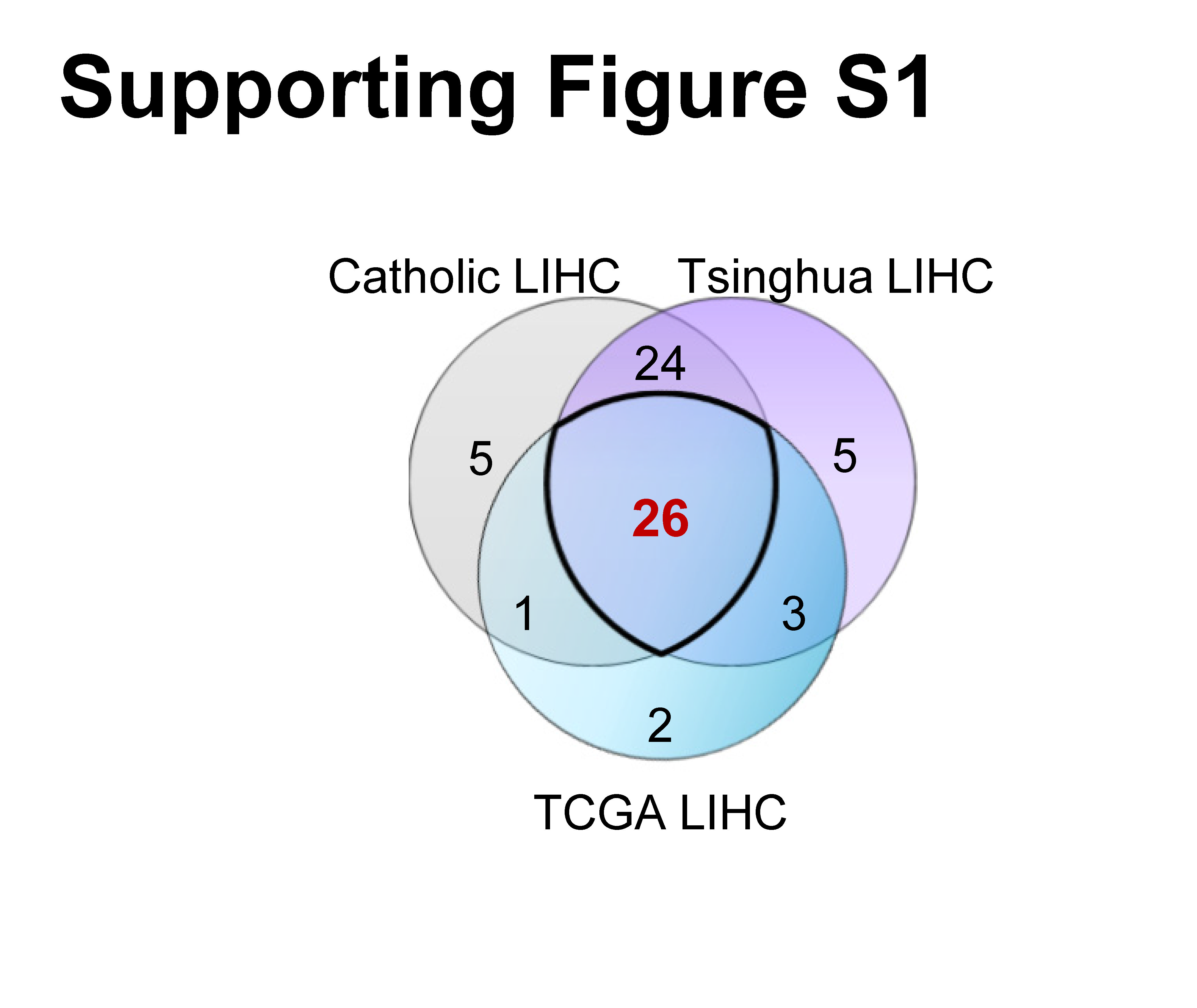

Supplement: Supplementary file 1 — Fig S1 [file CAM4-9-5459-s001.tiff]
