# Supplementary material for: Exosomal microRNA‐4661‐5p–based serum panel as a potential diagnostic biomarker for early‐stage hepatocellular carcinoma
Source: Cancer Med. 2020 Jun 14;9(15):5459–72. doi: 10.1002/cam4.3230 (PMC7402848; doi:10.1002/cam4.3230)
Supplement: Supplementary file 2 — Tabel S1 [file CAM4-9-5459-s002.doc]

**Supplementary Table S1. List of primer sequences used in qRT-PCR**

|  | Gene | | Accession No. | | Mature sequence | | Primer sequence |
| --- | --- | --- | --- | --- | --- | --- | --- |
| *hsa-miR-25-3p* | | MIMAT0000081 | | 5’-CAUUGCACUUGUCUCGGUCUGA -3’ | | 5'-CATTGCACTTGTCTCGGTCTGA-3' | |
| *hsa-miR-140-3p* | | MIMAT0004597 | | 5’-UACCACAGGGUAGAACCACGG-3’ | | 5'-TACCACAGGGTAGAACCACGG-3' | |
| *hsa-miR-423-3p* | | MIMAT0001340 | | 5’-AGCUCGGUCUGAGGCCCCUCAGU-3’ | | 5'-AGCTCGGTCTGAGGCCCCTCAGT-3' | |
| *hsa-miR-1269a* | | MIMAT0005923 | | 5’-CUGGACUGAGCCGUGCUACUGG-3’ | | 5'-CTGGACTGAGCCGTGCTACTGG-3' | |
| *hsa-miR-4661-5p* | | MIMAT0019729 | | 5’-AACUAGCUCUGUGGAUCCUGAC-3’ | | 5'-AACTAGCTCTGTGGATCCTGAC-3' | |
| *hsa-miR-4746-5p* | | MIMAT0019880 | | 5’-CCGGUCCCAGGAGAACCUGCAGA-3’ | | 5'-CCGGTCCCAGGAGAACCTGCAGA-3' | |
| *hsa-miR-1228-3p* | | MIMAT0005583 | | 5’-UCACACCUGCCUCGCCCCCC-3’ | | 5'-TCACACCTGCCTCGCCCCCC-3' | |

qRT-PCR, quantitative real-time polymerase chain reaction; miR, microRNA
