# Supplementary material for: Exosomal microRNA‐4661‐5p–based serum panel as a potential diagnostic biomarker for early‐stage hepatocellular carcinoma
Source: Cancer Med. 2020 Jun 14;9(15):5459–72. doi: 10.1002/cam4.3230 (PMC7402848; doi:10.1002/cam4.3230)
Supplement: Supplementary file 3 — Table S2 [file CAM4-9-5459-s003.doc]

**Supplementary Table S2. List of 26 miRs and clinicopathological score of each miR in** TCGA LIHC cohort

|  | Overall survival | | | Disease free survival | | |  |  |  |
| --- | --- | --- | --- | --- | --- | --- | --- | --- | --- |
| miRNA | P value | Hazard Ratio (logrank) | 95% CI of ratio | P value | Hazard Ratio (logrank) | 95% CI of ratio | OS/DFS | wHCC | Clinicopathologic  score |
| hsa-mir-1226 | 0.007 | 1.611 | 1.138 to 2.28 | 0.0053 | 1.514 | 1.122 to 2.043 | 1 |  | 1 |
| hsa-mir-1269a | 0.0463 | 1.423 | 1.006 to 2.013 | 0.1708 | 1.228 | 0.9139 to 1.651 | 1 | 1 | 2 |
| hsa-mir-1276 | 0.2892 | 1.206 | 0.8526 to 1.705 | 0.6615 | 1.068 | 0.7945 to 1.435 |  | 1 | 1 |
| hsa-mir-140 | 0.8353 | 1.037 | 0.7335 to 1.467 | 0.033 | 1.377 | 1.024 to 1.852 | 1 | 1 | 2 |
| hsa-mir-183 | 0.1831 | 1.263 | 0.892 to 1.788 | 0.6369 | 1.073 | 0.7978 to 1.444 |  | 1 | 1 |
| hsa-mir-185 | 0.8831 | 1.026 | 0.7257 to 1.451 | 0.5101 | 0.9059 | 0.674 to 1.218 |  | 1 | 1 |
| hsa-mir-190b | 0.2505 | 1.224 | 0.8646 to 1.732 | 0.3554 | 1.149 | 0.8549 to 1.545 |  | 1 | 1 |
| hsa-mir-21 | 0.0564 | 1.395 | 0.9845 to 1.976 | 0.1507 | 1.241 | 0.9225 to 1.669 |  | 1 | 1 |
| hsa-mir-25 | 0.0857 | 1.352 | 0.9546 to 1.914 | 0.0004 | 1.694 | 1.258 to 2.281 | 1 | 1 | 2 |
| hsa-mir-301a | 0.0016 | 1.746 | 1.233 to 2.473 | 0.0004 | 1.685 | 1.25 to 2.271 | 1 |  | 1 |
| hsa-mir-301b | 0.0683 | 1.377 | 0.9728 to 1.95 | 0.2497 | 1.189 | 0.8842 to 1.599 |  | 1 | 1 |
| hsa-mir-30d | 0.3379 | 0.8447 | 0.5973 to 1.195 | 0.8086 | 0.9643 | 0.7176 to 1.296 |  |  | 0 |
| hsa-mir-3144 | 0.7109 | 0.9367 | 0.6623 to 1.325 | 0.7236 | 1.055 | 0.7849 to 1.417 |  | 1 | 1 |
| hsa-mir-3591 | 0.2235 | 1.238 | 0.8745 to 1.752 | 0.1124 | 1.267 | 0.9417 to 1.705 |  | 1 | 1 |
| hsa-mir-3677 | <0.0001 | 2.381 | 1.682 to 3.371 | 0.0027 | 1.564 | 1.158 to 2.111 | 1 |  | 1 |
| hsa-mir-423 | 0.0021 | 1.725 | 1.218 to 2.442 | 0.0008 | 1.648 | 1.224 to 2.22 | 1 | 1 | 2 |
| hsa-mir-4326 | 0.8558 | 1.033 | 0.7299 to 1.461 | 0.4972 | 1.108 | 0.8241 to 1.488 |  |  | 0 |
| hsa-mir-454 | 0.0076 | 1.598 | 1.129 to 2.264 | 0.0013 | 1.614 | 1.198 to 2.175 | 1 |  | 1 |
| hsa-mir-4661 | 0.0076 | 1.596 | 1.126 to 2.262 | 0.6416 | 1.073 | 0.7974 to 1.443 | 1 | 1 | 2 |
| hsa-mir-4677 | 0.6098 | 1.094 | 0.7736 to 1.547 | 0.0349 | 1.373 | 1.021 to 1.846 | 1 |  | 1 |
| hsa-mir-4746 | 0.0049 | 1.644 | 1.161 to 2.328 | 0.0376 | 1.366 | 1.015 to 1.839 | 1 | 1 | 2 |
| hsa-mir-500a | 0.0659 | 1.38 | 0.974 to 1.956 | 0.0028 | 1.564 | 1.161 to 2.106 | 1 |  | 1 |
| hsa-mir-500b | 0.1718 | 1.27 | 0.8971 to 1.799 | 0.0977 | 1.281 | 0.9506 to 1.726 |  |  | 0 |
| hsa-mir-501 | 0.0493 | 1.416 | 1.001 to 2.003 | 0.0823 | 1.298 | 0.9648 to 1.746 | 1 |  | 1 |
| hsa-mir-532 | 0.2325 | 1.233 | 0.8709 to 1.745 | 0.0639 | 1.322 | 0.9834 to 1.776 |  | 1 | 1 |
| hsa-mir-769 | 0.0049 | 1.646 | 1.163 to 2.329 | 0.1581 | 1.235 | 0.9183 to 1.662 | 1 |  | 1 |
| hsa-mir-93 | 0.4518 | 1.141 | 0.8062 to 1.616 | 0.016 | 1.435 | 1.067 to 1.93 | 1 |  | 1 |

miR, microRNA; wHCC; well-differentiated HCC
