# Supplementary material for: Exosomal microRNA‐4661‐5p–based serum panel as a potential diagnostic biomarker for early‐stage hepatocellular carcinoma
Source: Cancer Med. 2020 Jun 14;9(15):5459–72. doi: 10.1002/cam4.3230 (PMC7402848; doi:10.1002/cam4.3230)
Supplement: Supplementary file 4 — Tabel S3 [file CAM4-9-5459-s004.doc]

**Supplementary Table S3. Areas under the curves of serum AFP and derived serum panels for diagnosing HCC**

|  | N,CH,LC vs HCC | |  | CH,LC vs HCC | |  | N,CH,LC vs mUICC1&2 | |  | CH,LC vs mUICC1&2 | |  | N,CH,LC vs mUICC1 | |  | CH,LC vs mUICC1 | |
| --- | --- | --- | --- | --- | --- | --- | --- | --- | --- | --- | --- | --- | --- | --- | --- | --- | --- |
|  | AUC | 95% CI |  | AUC | 95% CI |  | AUC | 95% CI |  | AUC | 95% CI |  | AUC | 95% CI |  | AUC | 95% CI |
| AFP | 0.704 | 0.625-0.782 |  | 0.597 | 0.501-0.692 |  | 0.597 | 0.497-0.696 |  | 0.540 | 0.439-0.639 |  | 0.541 | 0.429-0.651 |  | 0.604 | 0.494-0.707 |
| AFP+miR-25 | 0.790 | 0.723-0.857 |  | 0.794 | 0.718-0.869 |  | 0.697 | 0.606-0.787 |  | 0.714 | 0.613-0.814 |  | 0.735 | 0.635-0.834 |  | 0.812 | 0.716-0.907 |
| AFP+miR-1269a | 0.872 | 0.820-0.924 |  | 0.887 | 0.833-0.940 |  | 0.831 | 0.760-0.902 |  | 0.854 | 0.782-0.925 |  | 0.83 | 0.753-0.906 |  | 0.868 | 0.794-0.941 |
| AFP+miR-4661 | 0.921 | 0.883-0.959 |  | 0.921 | 0.879-0.961 |  | 0.911 | 0.862-0.959 |  | 0.910 | 0.857-0.962 |  | 0.925 | 0.874-0.975 |  | 0.929 | 0.877-0.980 |
| AFP+miR-4746 | 0.741 | 0.665-0.817 |  | 0.520 | 0.425-0.615 |  | 0.662 | 0.568-0.756 |  | 0.365 | 0.251-0.477 |  | 0.623 | 0.504-0.741 |  | 0.699 | 0.574-0.824 |
| miR-25+miR-1269a | 0.850 | 0.793-0.907 |  | 0.872 | 0.813-0.929 |  | 0.834 | 0.765-0.902 |  | 0.853 | 0.782-0.924 |  | 0.81 | 0.728-0.890 |  | 0.832 | 0.748-0.915 |
| miR-25+miR-4661 | 0.919 | 0.880-0.957 |  | 0.918 | 0.876-0.959 |  | 0.923 | 0.877-0.967 |  | 0.926 | 0.879-0.972 |  | 0.931 | 0.883-0.978 |  | 0.933 | 0.885-0.981 |
| miR-25+miR-4746 | 0.790 | 0.720-0.860 |  | 0.826 | 0.758-0.893 |  | 0.713 | 0.615-0.810 |  | 0.749 | 0.649-0.848 |  | 0.707 | 0.596-0.817 |  | 0.731 | 0.611-0.850 |
| miR-1269a+miR-4661 | 0.918 | 0.879-0.956 |  | 0.918 | 0.876-0.959 |  | 0.91 | 0.861-0.958 |  | 0.911 | 0.859-0.963 |  | 0.925 | 0.874-0.975 |  | 0.927 | 0.875-0.978 |
| miR-1269a+miR-4746 | 0.874 | 0.821-0.927 |  | 0.918 | 0.873-0.962 |  | 0.856 | 0.791-0.920 |  | 0.905 | 0.846-0.962 |  | 0.837 | 0.760-0.913 |  | 0.883 | 0.808-0.957 |
| miR-4661+miR-4746 | 0.942 | 0.908-0.975 |  | 0.948 | 0.915-0.981 |  | 0.945 | 0.909-0.979 |  | 0.951 | 0.914-0.986 |  | 0.947 | 0.907-0.987 |  | 0.954 | 0.916-0.992 |

N, normal subjects; CH, chronic hepatitis; LC, liver cirrhosis; HCC, hepatocelullar carcinoma; mUICC, modified Union for International Cancer Control; AFP, alpha-fetoprotein; miR, microRNA; AUC, Area under the curve
