# Supplementary material for: Exosomal microRNA‐4661‐5p–based serum panel as a potential diagnostic biomarker for early‐stage hepatocellular carcinoma
Source: Cancer Med. 2020 Jun 14;9(15):5459–72. doi: 10.1002/cam4.3230 (PMC7402848; doi:10.1002/cam4.3230)
Supplement: Supplementary file 5 — Table S4 [file CAM4-9-5459-s005.doc]

**Supplementary** Table S4. Cut-off values of the serum exo-miR-4661-5p-based panels

|  | AFP+ miR-4661-5p | miR-4661-5p+miR-4746-5p |
| --- | --- | --- |
| HCC vs Non tumor | 0.6049 | 0.3788 |
| mUICC I&II vs Non tumor | 0.1455 | 0.4581 |
| mUICC I vs Non tumor | 0.5589 | 0.3629 |
| HCC vs CH&LC | 0.7431 | 0.2357 |
| mUICC I&II vs CH&LC | 0.2074 | 0.2302 |
| mUICC I vs CH&LC | 0.6317 | 0.3366 |

Exo-miR, exosomal microRNA; AFP, alpha-fetoprotein; miR, microRNA; HCC, hepatocellular carcinoma; CH, chronic hepatitis; LC, liver cirrhosis; mUICC, modified Union for International Cancer Control
